# An overlooked oxidation mechanism of toluene: computational predictions and experimental validations†

**DOI:** 10.1039/d3sc03638c

**Published:** 2023-10-27

**Authors:** Zihao Fu, Fangfang Ma, Yuliang Liu, Chao Yan, Dandan Huang, Jingwen Chen, Jonas Elm, Yuanyuan Li, Aijun Ding, Lukas Pichelstorfer, Hong-Bin Xie, Wei Nie, Joseph S. Francisco, Putian Zhou

**Affiliations:** a Key Laboratory of Industrial Ecology and Environmental Engineering (Ministry of Education), School of Environmental Science and Technology, Dalian University of Technology Dalian 116024 China hbxie@dlut.edu.cn; b Joint International Research Laboratory of Atmospheric and Earth System Sciences, School of Atmospheric Sciences, Nanjing University Nanjing 210023 China niewei@nju.edu.cn; c State Environmental Protection Key Laboratory of Formation and Prevention of Urban Air Pollution Complex, Shanghai Academy of Environmental Sciences Shanghai China; d Department of Chemistry, iClimate, Aarhus University Langelandsgade 140 DK-8000 Aarhus C Denmark; e Institute for Atmospheric and Earth System Research/Physics, Faculty of Science, University of Helsinki P. O. Box 64 FIN-00014 Helsinki Finland putian.zhou@helsinki.fi; f pi-numerics Wallbachsiedlung 28 5202 Neumarkt am W. Austria; g Department of Earth and Environmental Science, University of Pennsylvania Philadelphia PA USA 19104-6316 frjoseph@sas.upenn.edu

## Abstract

Secondary organic aerosols (SOAs) influence the Earth's climate and threaten human health. Aromatic hydrocarbons (AHs) are major precursors for SOA formation in the urban atmosphere. However, the revealed oxidation mechanism dramatically underestimates the contribution of AHs to SOA formation, strongly suggesting the importance of seeking additional oxidation pathways for SOA formation. Using toluene, the most abundant AHs, as a model system and the combination of quantum chemical method and field observations based on advanced mass spectrometry, we herein demonstrate that the second-generation oxidation of AHs can form novel epoxides (TEPOX) with high yield. Such TEPOX can further react with H_2_SO_4_ or HNO_3_ in the aerosol phase to form less-volatile compounds including novel non-aromatic and ring-retaining organosulfates or organonitrates through reactive uptakes, providing new candidates of AH-derived organosulfates or organonitrates for future ambient observation. With the newly revealed mechanism, the chemistry-aerosol box modeling revealed that the SOA yield of toluene oxidation can reach up to 0.35, much higher than 0.088 based on the original mechanism under the conditions of pH = 2 and 0.1 ppbv NO. This study opens a route for the formation of reactive uptake SOA precursors from AHs and significantly fills the current knowledge gap for SOA formation in the urban atmosphere.

## Abstract 2

It provides a new pathway for the SOA formation from toluene oxidation in the urban atmosphere.

## Graphical abstract

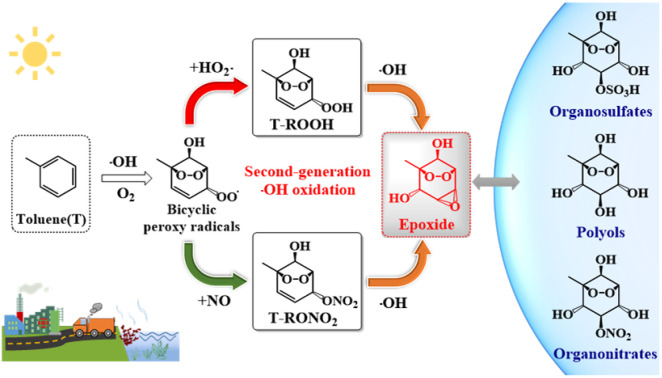

## Introduction

Secondary organic aerosols (SOAs) represent a major constituent of atmospheric aerosols,^1^ and impact human health and global climate.^2,3^ Gaseous organic compounds are potential SOA precursors, especially volatile organic compounds (VOCs). In the past 70 years, gas-phase oxidation of VOCs followed by condensation has been suggested to dominate SOA formation.^1^ Great efforts have been made to reveal the oxidation mechanism of VOCs, and identify the SOA precursors to build a quantitative relationship of gaseous organic compounds with SOA formation.^4,5^ However, atmospheric models based on current information consistently underestimate the global SOA budget.^6–8^ This underestimation of SOAs strongly suggests the importance of seeking additional pathways leading to SOA formation.

Increasing evidence suggests that multiphase chemistry caused by the formation of reactive uptake precursors (RUPs) in the oxidation of VOCs is an important pathway for SOA formation. For example, the reactive uptake of isoprene-derived epoxydiols (IEPOX) has been demonstrated to be a significant source of atmospheric SOAs globally.^9–12^ Most recently, it was suggested that even in polluted urban environments, RUPs of anthropogenic origin significantly contributed to SOA formation locally.^13–16^ However, the underlying sources and chemical identities of these anthropogenic RUPs remain unclear. Therefore, the identification of the mechanisms of RUP formation from anthropogenic VOC precursors is of great interest.

Aromatic hydrocarbons (AHs) comprise a significant fraction (up to 60%) of total VOCs in the urban atmosphere.^17^ The oxidation of AHs can significantly contribute to SOA formation with up to 50% in the urban atmosphere in eastern China.^18^ The most abundant AHs are monocyclic aromatic hydrocarbons (MAHs) such as benzene and toluene.^17^ The first-generation and multi-generation oxidation mechanisms of MAHs have previously been investigated.^19–29^ Particularly, the autoxidation mechanism which leads to the formation of highly oxygenated organic molecules (HOMs) has been identified for alkylbenzene.^22,23^ With the revealed mechanism, the condensation of the low-volatility HOMs and multiphase chemistry of glyoxal and methylglyoxal were found to be the main processes for SOA formation of MAH oxidation. However, considering these processes still underestimated the SOA yield,^30–33^ indicates the existence of missing MAH oxidation mechanisms and possible unidentified RUPs.

Here, we demonstrate that the second-generation oxidation of MAHs can produce a significant yield of epoxides, enhancing SOA production through reactive uptakes under low pH conditions, similar to the case of isoprene.^34,35^ We selected toluene (T) as the representative compound as it is the most abundant AH in the urban atmosphere.^20,36^ Specifically, the modeled system started with hydroperoxide T-ROOH and organonitrate T-RONO_2_, which are important first-generation products of toluene upon oxidation by OH.^19^ The formation of epoxides is revealed by quantum chemical calculations and kinetics modeling, which are supported by field observations. With the chemistry-aerosol SOSAA-Box model, the SOA yield is shown to increase substantially when the reactive uptake of epoxides is considered. This study presents a new route for RUP formation from AHs, guides the detection of novel AH-derived SOA precursors, and fills the current knowledge gap in SOA formation in the urban atmosphere.

## Materials and methods

### Global minimum search

The global minimum of T-ROOH and T-RONO_2_ was selected as the initial conformations for the study of the multi-generation oxidation mechanism. A similar scheme for the global minimum search has been employed in our previous studies.^37,38^ Briefly, *ab initio* molecular dynamics (AIMD) within the TURBOMOLE 6.5 program package^39^ was first performed to produce a range of conformations of T-ROOH and T-RONO_2_. Selected conformations from the AIMD run were then further optimized at the M06-2X/6-31+G(d,p) level of theory, followed by ROCBS-QB3 single-point energy calculations. The conformation with the lowest Gibbs free energy was identified as the global minimum of T-ROOH and T-RONO_2_ (see their structures in Fig. S1†).

### *Ab initio* electronic structure calculations

All electronic structure and energy calculations were performed using the GAUSSIAN 09 program package.^40^ The geometry optimizations and harmonic vibrational frequency calculations for reactants (R), pre-complexes (RCs), post-complexes (PCs), intermediates (IMs), transition states (TSs) and products involved in all reaction pathways were performed at the M06-2X/6-31+G(d,p) level of theory,^41^ followed by a higher level ROCBS-QB3 single-point energy calculation.^42^ The combination of M06-2X functional with the ROCBS-QB3 scheme has previously been used in studying the oxidation of AHs.^22,24,43–46^ Since reaction pathways with high reaction barriers contribute negligibly to the reaction kinetics, only low level energies of the species involved in the pathways were provided (shown in the ESI†) considering the computational costs. Values of T_1_ diagnostics for the TSs in all reaction pathways were less than the threshold value (0.045) for the open-shell systems,^47^ indicating that single-reference methods are well suited to describe the target systems. To check the wavefunction stability of RC, the keyword “stable” was used. When considering reactions in the aqueous phase, the SMD solvation model was employed to account for the water solvent effect.^48^ In addition, the proportion of different dissociation forms of the reactants involved in the aqueous phase under different pH conditions was calculated based on the p*K*_a_ values. Intrinsic reaction coordinate (IRC) calculations were performed to confirm the connection of each TS between designated local minima.

### Kinetics calculations

Reaction rate constants for the unimolecular reactions with a well-defined transition state as well as the competition between the uni- and biomolecular reactions were modeled using Rice–Ramsperger–Kassel–Marcus (RRKM)-master equation (ME) theory in the MESMER program.^49^ For ·OH-initiated reactions, RCs involved in the ·OH-addition reaction pathways were considered for all the kinetic calculations,^50–54^ since the ·OH-addition reaction is the dominant pathway. Reaction rate constants for the barrierless bimolecular reactions from R to RC in the OH-initiated reaction were calculated by combining the use of long-range transition state theory with a dispersion force potential and the inverse Laplace transformation (ILT) method.^49,55^ For the bimolecular reaction (alkyl radicals + O_2_), a constant value of 6.0 × 10^−12^ cm^3^ per molecule per s was used, which is similar to previous studies.^22,56–58^ N_2_ was used as the buffer gas. The average collisional activation/deactivation energy transfer of all the molecules is set to 200 cm^−1^ (Δ*E*_d_) per collision and the grain size is 50 cm^−1^. To explore the effects of Δ*E*_d_ and grain size on the results, we additionally run the simulations at other Δ*E*_d_ (150, 250 and 300 cm^−1^) and grain size (25 cm^−1^). The empirical method proposed by Gilbert and Smith was applied to estimate the Lennard-Jones parameters of intermediates (Table S2†).^59^ The theory for calculating the fractional yields of the main intermediates is presented in the ESI.† A one-dimensional unsymmetrical Eckart barrier was used to account for the tunneling effects in all the reaction rate constant calculations involving H-shift or H-abstraction.^60^

### Field observations

Ambient data of toluene and oxygenated organic molecules (OOMs) from aromatic oxidation were collected during the summer in Nanjing, a megacity in eastern China.^61^ Detailed description of this data has been presented in our previous study.^61^ Briefly, toluene was measured using a PTR-TOF-MS (Ionicon Analytik, TOF 1000 ultra),^62^ while OOMs were measured by using a nitrate-ion-based chemical ionization atmospheric pressure interface time-of-flight mass spectrometer (nitrate CI-APi-TOF), with a mass resolution of 8000–12 000 Th Th^−1^ (Th denotes Thomsons).^63,64^ The concentrations of OOMs were estimated *via*^65,66^1
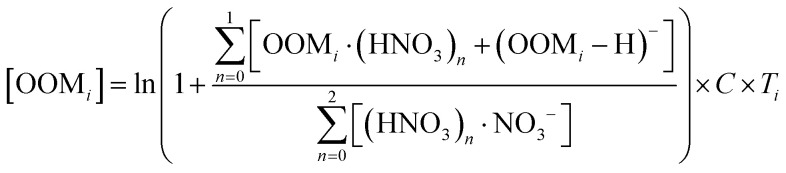
Here, [OOM_*i*_] is the concentration (molecules per cm^3^) of one OOM. First, we calibrated sulfuric acid (SA) by introducing a known amount of gaseous SA. The diffusion loss of SA was taken into account to obtain the calibration factor *C*. Then we used this factor *C* to calibrate the detected OOMs by assuming they have the same ionization efficiency as SA.^4,65^ Second, a mass-dependent transmission efficiency *T*_i_ of APi-TOF was inferred in a separate experiment by depleting the reagent ions with several perfluorinated acids.^67^

The primary RO_2_· (P_C7-Aro-RO_2__) is calculated as *k*_OH_ × [toluene] × [·OH], where *k*_OH_ is the reaction rate constant (5.0 × 10^−13^ cm^3^ per molecule per s) of toluene with ·OH,^18^ and [toluene] and [·OH] are the concentrations of toluene and ·OH, respectively. [·OH] was estimated from the concentration of SA ([SA]) (*via*eqn (2)).^68^2
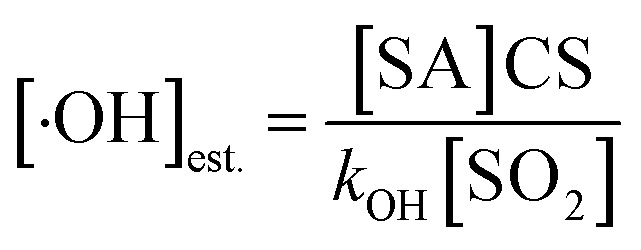
where the [SA] was measured by nitrate CI-APi-TOF; CS is the condensation sink, calculated based on the measurement of aerosol size distribution; [SO_2_] (SO_2_ concentration) was measured using a Thermo TEI 43i SO_2_ analyzer.

### Box modeling

The box model SOSAA-Box^69^ (model to simulate organic vapours, sulphuric acid and aerosols) was applied to simulate the effect of the new oxidation pathways of toluene on SOA mass yields. The chemistry scheme was first generated with the MCM v3.3.1 (Master Chemical Mechanism version 3.3.1)^70^ by selecting the following species: toluene and CH_4_. The reaction rates of the oxidation of SO_2_ by stabilized Criegee intermediate (sCI) radicals were increased to 7.0 × 10^−13^ cm^3^ per molecule per s from 7.0 × 10^−14^ cm^3^ per molecule per s as suggested in Boy *et al.*^71^ All considered reaction pathways are presented in the ESI.† The background particle size distributions (PSDs) representing the environmental conditions in typical cities refer to the data collected in Wu and Boor,^72^ in which all the measured PSDs have been fitted with three lognormal modes. For example, one sample of PSDs in Beijing measured by Massling *et al.*^73^ has been fitted to three modes in the range of 3 nm to 800 nm with geometric mean diameters of 5.7 nm, 32.8 nm, and 114.5 nm, geometric standard deviations of 1.33, 2.61, and 1.55, and the peak number concentrations of 615 molecules cm^−3^, 31 702 molecules cm^−3^, and 614 molecules cm^−3^, respectively (see Table S3† in Wu and Boor^72^). These PSDs were also applied and kept constant in this study to simulate the background aerosol environment. Therefore, nucleation and coagulation were not considered in the simulations.

In order to quantify how the oxidation products can contribute to SOA formation under different conditions and chemistry mechanisms, the condensation/evaporation processes of condensable organic vapors were simulated with the analytical predictor of condensation (APC) scheme modified from Jacobson.^74^ All the condensed organic compounds were considered to be well-mixed in the liquid phase. In this study, we have focused on the contribution of organic products, so the condensation of inorganic species is not considered. Each particle size was assumed to be internally mixed. The saturation vapor pressure (SVP) of the chemical species over a flat pure compound surface was obtained from the database in ARCA-Box,^75^ the SVP values of additional species in the new oxidation pathway were calculated using the SIMPOL method^76^ or *via* the EPI suite software (US EPA, 2012).^77^ The Raoult effect and Kelvin effect were included when calculating the SVP values over the particle surface. The activity coefficients were assumed to be one for all condensable vapors. Moreover, the method from eqn (17) in Jacobson^78^ was applied at each time step to constrain the mass of condensed vapors to not exceed the total available amount. The aqueous phase chemical reactions were calculated explicitly after condensation/evaporation processes when needed in the simulation cases. Other details for the model setup are presented in the ESI.†

## Results and discussion

### Formation of epoxides in the reactions of T-ROOH and T-RONO_2_ with ·OH

**Fig. 1:**
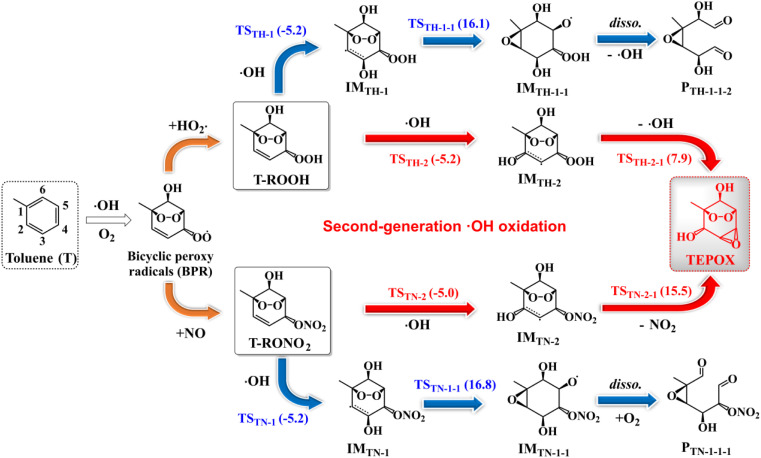
Reaction pathways of forming epoxides for reactions of T-ROOH and T-RONO_2_ with ·OH starting from toluene (T). The numbers (in kcal mol^−1^) near the arrows are zero-point corrected reaction energy barriers for the corresponding reactions at the ROCBS-QB3//M06-2X/6-31+G(d,p) level of theory. The labels TS_TH/TN-*m*_, IM_TH/TN-*m*_ and P_TH/TN-*m*_ represent the transition states, intermediates and products, respectively, where subscripts TH/TN were used to differentiate the reactions starting from T-ROOH/T-RONO_2_ + ·OH, respectively, and *m* presents different species.

By carefully considering all possible reaction pathways (Fig. S2–S5†) for the ·OH-initiated oxidation of T-ROOH and T-RONO_2_, we identify the reaction pathways for forming novel epoxides (Fig. 1), which can potentially contribute to SOA formation through multiphase reactions similar to IEPOX.^9–12^ Two types of epoxides are identified including ring-opening (here the ring refers to a six-membered ring) dicarbonyl epoxides (P_TH-1-1-2_ and P_TN-1-1-1_) and ring-retaining epoxides (TEPOX). Ring-opening epoxides are formed in a multi-step reaction mechanism that proceeds *via* a C-centered radical intermediate formed by ·OH addition to the α-site C-atom of the –COOH/CONO_2_ group. The formation mechanism of the ring-opening epoxides for the reaction of T-ROOH is slightly different from that for the reactions of T-RONO_2_. For the reactions of T-ROOH, the formed RO· from C-centered radicals intermediately dissociates to form P_TH-1-1-2_, but not IM_TH-1-1-1_*via* the lower reaction energy barriers (*E*_a_) (see detailed analysis in the ESI). However, the formed RO· from C-centered radical intermediates needs multiple steps to finally form P_TN-1-1-1_ for the reactions of T-RONO_2_. Differing from the ring-opening epoxides, ring-retaining TEPOX is formed *via* a two-step reaction mechanism that proceeds *via* ·OH addition to the β-site C-atom of the –COOH/CONO_2_ group, followed by a concerted O–O/O–N bond rupture and C–O–C cyclization.

The formation mechanism for ring-retaining TEPOX from T-ROOH and T-RONO_2_ is similar to that of IEPOX from organic hydroperoxide ISOPOOH and organonitrate ISOPONO_2_ formed from the oxidation of isoprene.^9,79^ In view of the molecular structure, the similar reaction mechanism should result from the fact that they contain similar <svg xmlns="http://www.w3.org/2000/svg" version="1.0" width="10.400000pt" height="16.000000pt" viewBox="0 0 10.400000 16.000000" preserveAspectRatio="xMidYMid meet"><metadata> Created by potrace 1.16, written by Peter Selinger 2001-2019 </metadata><g transform="translate(1.000000,15.000000) scale(0.011667,-0.011667)" fill="currentColor" stroke="none"><path d="M80 1160 l0 -40 40 0 40 0 0 -40 0 -40 40 0 40 0 0 -40 0 -40 40 0 40 0 0 -40 0 -40 40 0 40 0 0 -40 0 -40 40 0 40 0 0 -40 0 -40 40 0 40 0 0 -40 0 -40 40 0 40 0 0 80 0 80 -40 0 -40 0 0 40 0 40 -40 0 -40 0 0 40 0 40 -40 0 -40 0 0 40 0 40 -40 0 -40 0 0 40 0 40 -40 0 -40 0 0 40 0 40 -80 0 -80 0 0 -40z M560 520 l0 -40 -40 0 -40 0 0 -40 0 -40 -40 0 -40 0 0 -40 0 -40 -40 0 -40 0 0 -40 0 -40 -40 0 -40 0 0 -40 0 -40 -40 0 -40 0 0 -40 0 -40 -40 0 -40 0 0 -40 0 -40 80 0 80 0 0 40 0 40 40 0 40 0 0 40 0 40 40 0 40 0 0 40 0 40 40 0 40 0 0 40 0 40 40 0 40 0 0 40 0 40 40 0 40 0 0 80 0 80 -40 0 -40 0 0 -40z"/></g></svg> C=CH–C(–OOH/ONO_2_) <svg xmlns="http://www.w3.org/2000/svg" version="1.0" width="10.400000pt" height="16.000000pt" viewBox="0 0 10.400000 16.000000" preserveAspectRatio="xMidYMid meet"><metadata> Created by potrace 1.16, written by Peter Selinger 2001-2019 </metadata><g transform="translate(1.000000,15.000000) scale(0.011667,-0.011667)" fill="currentColor" stroke="none"><path d="M480 1160 l0 -40 -40 0 -40 0 0 -40 0 -40 -40 0 -40 0 0 -40 0 -40 -40 0 -40 0 0 -40 0 -40 -40 0 -40 0 0 -40 0 -40 -40 0 -40 0 0 -80 0 -80 40 0 40 0 0 40 0 40 40 0 40 0 0 40 0 40 40 0 40 0 0 40 0 40 40 0 40 0 0 40 0 40 40 0 40 0 0 40 0 40 40 0 40 0 0 40 0 40 40 0 40 0 0 40 0 40 -80 0 -80 0 0 -40z M80 480 l0 -80 40 0 40 0 0 -40 0 -40 40 0 40 0 0 -40 0 -40 40 0 40 0 0 -40 0 -40 40 0 40 0 0 -40 0 -40 40 0 40 0 0 -40 0 -40 80 0 80 0 0 40 0 40 -40 0 -40 0 0 40 0 40 -40 0 -40 0 0 40 0 40 -40 0 -40 0 0 40 0 40 -40 0 -40 0 0 40 0 40 -40 0 -40 0 0 40 0 40 -40 0 -40 0 0 40 0 40 -40 0 -40 0 0 -80z"/></g></svg> structural units, which act as the reactive core for forming the epoxides. It is noteworthy that the reaction energy barriers (*E*_a_) for the formation of TEPOX from T-ROOH are much lower than that from T-RONO_2_, which resembles the formation of IEPOX from ISOPOOH and ISOPONO_2_.^79^

**Fig. 2:**
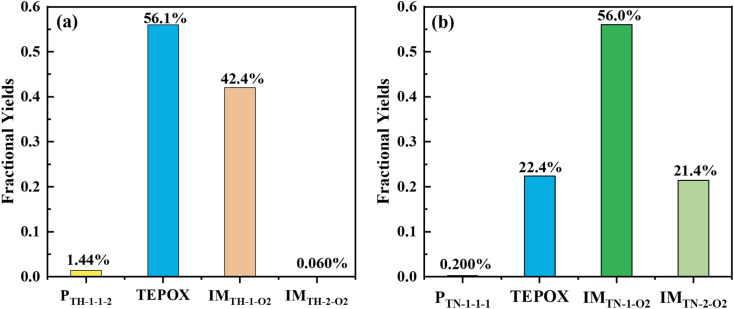
Calculated fractional yields of main intermediates and products in the reactions of T-ROOH (a) and T-RONO_2_ (b) initiated by ·OH at 298 K and 1 atm. The labels IM_TH/TN-*m*-O_2__ and P_TH/TN-*m*_ represent the peroxy radicals and products, respectively, where subscripts TH/TN were used to differentiate the reactions starting from T-ROOH/T-RONO_2_ + ·OH, respectively, and *m* presents different species.

Similar to a previous study,^80^ by considering all possible competitive reaction pathways (T-ROOH + ·OH → IM_TH-1_/IM_TH-2_ → IM_TH-1-1_/P_TH2-1_ and IM_TH-1_/IM_TH-2_ + O_2_ → IM_TH-1-O_2__/IM_TH-2-O_2__), the fractional yields of ring-opening epoxides (P_TH-1-1-2_) and ring-retaining TEPOX (P_TH2-1_) are calculated to be 1.44% and 56.1% for the reaction of T-ROOH with ·OH, respectively (Fig. 2, details in Fig. S6†). Therefore, epoxides, mainly consisting of ring-retaining TEPOX, are important products for the reactions of T-ROOH with ·OH. We noted that previous studies found that the yields of epoxides are low for the reactions of alkoxy radicals produced in the first-generation oxidation of AHs.^44,45,81,82^ To the best of our knowledge, this is the first time to illustrate that ring-retaining epoxides (TEPOX) can be formed in high yields in the second-generation oxidation of toluene, similar to that of isoprene oxidation. In addition, the ring-opening epoxides also have a considerable yield (1.44%), presenting a novel mechanism for the formation of ring-opening epoxides in the atmosphere. Different from the reactions of T-ROOH with ·OH, the calculated fractional yield of ring-retaining TEPOX (22.4%) from the reaction of T-RONO_2_ with ·OH (Fig. 2) is low based on the favorable reaction pathways (T-RONO_2_ + OH → IM_TN-1_/IM_TN-2_ → IM_TN-1-1_/P_TN-2-1_ and IM_TN-1_/IM_TN-2_ + O_2_ → IM_TN-1-O_2__/IM_TN-2-O_2__). The lower yield of TEPOX results from its corresponding high unimolecular reaction energy barrier (15.5 kcal mol^−1^). A previous study on the oxidation of isoprene found that the yield of IEPOX from the reaction of ISOPOOH with ·OH is much higher than that from the reaction of ISOPONO_2_ with ·OH.^79^ This is consistent with our findings for toluene here. In addition, the yield (0.200%) of ring-opening epoxides from the T-RONO_2_ with ·OH is also lower than that (1.44%) of the corresponding reactions of T-ROOH with ·OH.

Besides TEPOX, peroxy radicals also have high yields in the reactions of T-ROOH and T-RONO_2_ initiated by ·OH, presenting another main oxidation pathway. For the reaction of T-ROOH, peroxy radicals are mainly formed from the reactions of C-centered IM_TH-1_ radicals with O_2_. The yield of the formed peroxy radicals IM_TH-1-O_2__ is 42.4%. For the reaction of T-RONO_2_, peroxy radicals are formed from the C-centered radicals IM_TN-1_ and IM_TN-2_. The yields of the formed IM_TN-1-O_2__ and IM_TN-2-O_2__ are 56.0% and 21.4%, respectively. These peroxy radicals can subsequently react with NO or HO_2_· to form organonitrates, hydroperoxides and alkoxy radicals. The formed alkoxy radicals eventually produce a range of dicarbonyl products including C_3_H_4_O_3_, C_4_H_6_O_3_, C_4_H_6_O_5_, C_4_H_6_NO_5_, C_7_H_9_NO_8_ and methylglyoxal (see details in Fig. S3 and S5†). In addition, we found that the selection of Δ*E*_d_ (from 150 to 300 cm^−1^) and grain size have little effect on the yields of the important species mentioned above (Table S3†).

### Comparison with recent laboratory studies

The main atmospheric oxidation pathways and products of the ·OH-initiated reactions of T-ROOH and T-RONO_2_ are summarized in Fig. S7.† Overall, the main products include C_7_H_10_O_5_, C_3_H_4_O_3_, C_4_H_6_O_3_, C_4_H_6_NO_5_, C_7_H_9_NO_8_ and methylglyoxal, some of which (C_7_H_10_O_5_, C_4_H_6_O_3_ and C_7_H_9_NO_8_) have been detected in the chamber experiments of toluene oxidation performed by Zaytsev *et al.*^26^ More importantly, Zaytsev *et al.*^26^ suggested that C_7_H_10_O_5_ is a mixture of first- and second-generation oxidation products of toluene, consistent with our finding that the molecular formula corresponds to T-ROOH (first-generation products) and ring-retaining TEPOX (second-generation products). The evidence from these experiments further corroborates our mechanistic findings.

### Supporting evidence from field observations

We further conducted field observations at the Station for Observing Regional Processes of the Earth System (SORPES)^83^ during the summer of 2019 in Nanjing, China. A nitrate CI-Api-TOF was employed to detect the oxidation products of toluene, especially ring-retaining TEPOX in the real atmosphere. Most molecules identified in our revealed mechanism can be observed in the real atmosphere, including both the key oxidation products of C_7_H_9_NO_6_, C_7_H_10_O_5_ and C_7_H_9_NO_8_, and the fragmentation products (C_4_H_5_NO_6_, C_4_H_6_O_5_, C_4_H_6_O_3_ and C_3_H_4_O_3_). The product molecules C_7_H_9_NO_6_, C_7_H_10_O_5_ and C_7_H_9_NO_8_ that are not fragmented have a double-bond-equivalent (DBE) of 3, suggesting they were formed *via* ·OH attacking the benzene ring of toluene at daytime.^18^

**Fig. 3:**
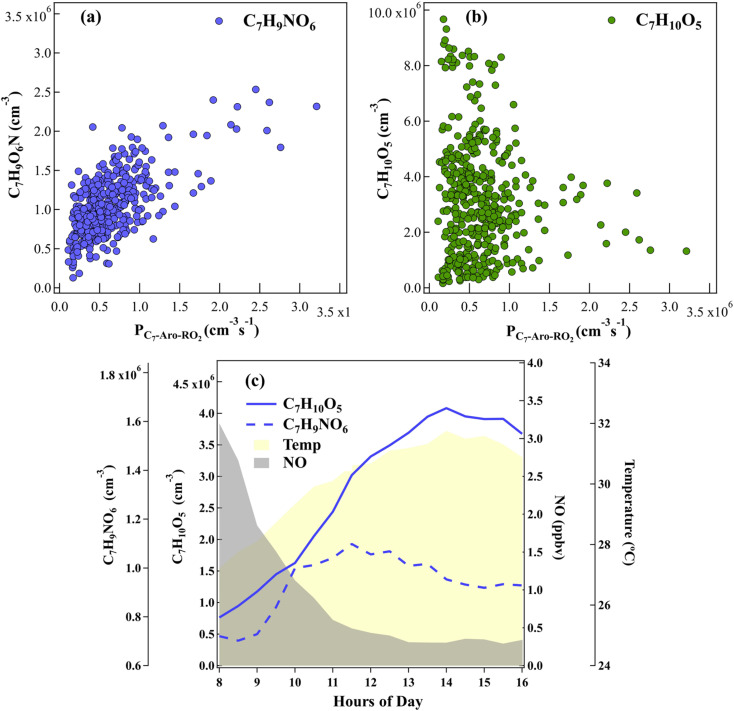
Ambient observation of toluene oxidation products of C_7_H_10_O_5_ and C_7_H_9_NO_6_. (a) Correlation of C_7_H_9_NO_6_ with a production rate of RO_2_ (P_C7 Aro-RO_2__) from ·OH-initiated oxidation of toluene during daytime, (b) correlation of C_7_H_10_O_5_ with the P_C7-Aro-RO_2__ during daytime, and (c) variation of C_7_H_10_O_5_, C_7_H_9_NO_6_, NO and temperature at daytime during the field observation campaign.

As shown in Fig. 3a, the observed C_7_H_9_NO_6_ correlates with the primary RO_2_· (P_C7-Aro-RO_2__) from the ·OH-initiated oxidation of toluene. Therefore, C_7_H_9_NO_6_ should correspond to T-RONO_2_ and is probably a first-generation product of toluene oxidation. However, we cannot determine whether C_7_H_10_O_5_ is T-ROOH, ring-retaining TEPOX or both directly from its elemental formula, since the mass spectrometry observations cannot distinguish molecular structures. As proposed above, the ring-retaining TEPOX molecule is a second-generation product, while T-ROOH is a first-generation product. Therefore, we infer the attribution of C_7_H_10_O_5_ by their distinctive diurnal variation patterns. As shown in Fig. 3b, there is no correlation between C_7_H_10_O_5_ and P_C7-Aro-RO_2__. More importantly, the daytime peak of C_7_H_10_O_5_ was around 14:00-15:00, well after the possible first-generation product C_7_H_9_NO_6_ (10:00–11:00) (Fig. 3c). Therefore, it is more likely that C_7_H_10_O_5_ is mainly composed of second-generation products (*i.e.* ring-retaining IEPOX), although some first-generation products may also be present in the morning. This is consistent with the previous lab study that C_7_H_10_O_5_ is a mixture of first- and second-generation products for the oxidation of toluene.^26^ Overall, the field observations suggest that a significant amount of ring-retaining TEPOX exists in this suburban environment.

### Box modelling

**Fig. 4:**
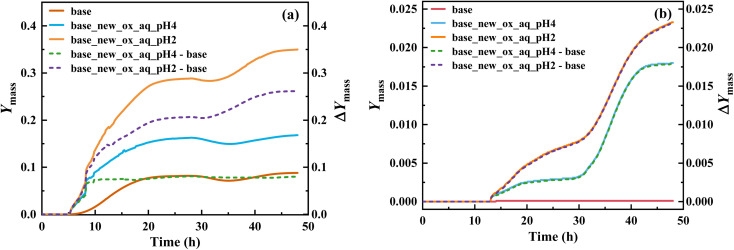
SOA mass yields (*Y*_mass_) for toluene oxidation based on the original mechanism (base) and new mechanism (base-new) and improved SOA mass yields (Δ*Y*_mass_) caused by the consideration of the new mechanism at pH = 2 and pH = 4 as a function of reaction time, under low NO concentration (0.1 ppbv) (a) and high NO concentration (5 ppbv) (b) conditions.

Implementing this new mechanism of T-ROOH and T-RONO_2_ initiated by ·OH, a SOSAA-Box model^69^ simulation shows that SOA yield significantly increases by 0.26 and 0.080 at pH = 2 and pH = 4 (Fig. 4a), respectively, when low NO concentration (*e.g.*, 0.1 ppbv) is considered. Even under the conditions of high NO concentration (*e.g.*, 5 ppbv), the SOA yield can increase by 0.023 and 0.018 at pH = 2 and pH = 4 (Fig. 4b), respectively. By analyzing the contribution of species to SOA, the TEPOX takes a high percentage (51.92%) at the condition of pH = 2 and 0.1 ppbv NO (see details in the ‘Analysis of sensitivity simulations’ part and Fig. S10 in the ESI†). This is consistent with its high fractional yields (56.1% for T-ROOH and 22.4% for T-RONO_2_) from the kinetic calculations. With increasing the pH and NO concentration, the contribution of TEPOX to SOA formation decreases, similar to the case of IEPOX.^34,35^ In addition, high SOA yield in low pH should mainly result from a high reaction rate of TEPOX (see box modeling details and sensitivity analysis in the ESI†). Therefore, this study uncovers a new mechanism for the formation of reactive uptake precursors that eventually connects gas-phase toluene oxidation to the SOA formation in an urban atmosphere, especially at low pH and low NO concentration.

## Atmospheric implication and conclusions

Our theoretical study and field observation reveal that ·OH-initiated oxidation of T-ROOH and T-RONO_2_, an important second-generation oxidation process of toluene, lead to the formation of ring-retaining TEPOX, and a range of dicarbonyl products. The formation of ring-retaining TEPOX, which resembles the formation of IEPOX from isoprene,^9,10,79^ has not been previously recognized. The formed TEPOX can form ring-retaining and non-aromatic organosulfates, organonitrates or polyols *via* acid-catalyzed ring-opening reactions once partitioned into the aerosol phase (see details in Figure S8†-9), similar to the heterogeneous reactions of the well-characterized IEPOX.^11,35,84^ Therefore, the identified TEPOX is a novel reactive uptake precursor for SOA formation. It should be noted that only aromatic organosulfates have been detected in the ambient particles *via* target analysis.^85–87^ This study suggests the existence of non-aromatic and ring-retaining AHs-derived organosulfates and organonitrates, which should be further investigated in future atmospheric measurements.

The revealed mechanism can significantly lead to the SOA increase for toluene oxidation, filling the SOA gap between experiment and model prediction under the conditions of low pH and low NO concentration, especially since the frequency of low-NO conditions has increased significantly in recent years due to NOx emission controls.^88^ Additionally, It is known that other AHs, especially MAHs, can form AHs-derived hydroperoxides and organonitrates in atmospheric oxidation.^26^ Accordingly, the oxidation of other AHs could also lead to the formation of epoxides through a similar pathway as toluene oxidation, which could significantly enhance SOA formation *via* reactive uptakes. More importantly, the present findings fill a gap in mechanistic chemical insight between measured and simulated SOA for AH oxidation, thereby, warranting future studies on the global contribution of this new mechanism to SOA formation.

## Data availability

The ESI† contains the details of box modelling; computational details for fractional yield calculation; comparison of the formation of P_TH-1-1-2_ and IM_TH-1-1-1_; discussion about the energies of RCs and TSs; all considered reaction pathways for the reactions of T-ROOH/RONO_2_ + ·OH; Lennard-Jones parameters of intermediates used in the MESMER simulations; effects of selection of Δ*E*_d_ and grain size on the yields of important species, proportion of different dissociation forms of TEPOX as a function of pH; cartesian coordinates and electronic energies.

## Author contributions

H. B. X. and J. S. F. designed the study. W. N., Y. L. L. and Y. Y. L. performed the field observation and analyzed the data. P. T. Z. and L. P. performed box model simulation. Z. H. F. and F. F. M. performed the quantum chemical calculation. H. B. X., J. S. F., C. Y., D. D. H. J. W. C., J. E., A. J. D., W. N., P. T. Z., Z. H. F. and F. F. M. wrote the manuscript. All coauthors participated in relevant scientific discussions and commented on the manuscript.

## Conflicts of interest

The authors declare no competing financial interest.

## Supplementary Material

SC-014-D3SC03638C-s001

SC-014-D3SC03638C-s002

SC-014-D3SC03638C-s003
