# Supplementary material for: An overlooked oxidation mechanism of toluene: computational predictions and experimental validations
Source: Chem Sci. 2023 Oct 27;14(45):13050–9. doi: 10.1039/d3sc03638c (PMC10664553; doi:10.1039/d3sc03638c)
Supplement: SC-014-D3SC03638C-s001 [file SC-014-D3SC03638C-s001.pdf]

Electronic Supplementary Material (ESI) for Chemical Science.
This journal is © The Royal Society of Chemistry 2023

Supporting Information

Overlooked Oxidation Mechanism of Toluene: Computational Predictions and Experimental Validations

Zihao Fu[1#], Fangfang Ma[1#], Yuliang Liu[2], Chao Yan[2], Dandan Huang[3], Jingwen Chen[1], Jonas Elm[4], Yuanyuan Li[2], Aijun Ding[2], Lukas Pichelstorfer[5,6], Hong-Bin Xie[1*], Wei Nie[2*], Joseph S. Francisco[7*], Putian Zhou[5*]

[1]Key Laboratory of Industrial Ecology and Environmental Engineering (Ministry of Education), School of Environmental Science and Technology, Dalian University of Technology, Dalian 116024, China

[2]Joint International Research Laboratory of Atmospheric and Earth System Sciences, School of Atmospheric Sciences, Nanjing University, Nanjing 210023, China

[3]State Environmental Protection Key Laboratory of Formation and Prevention of Urban Air Pollution Complex, Shanghai Academy of Environmental Sciences, Shanghai, China

[4]Department of Chemistry, iClimate, Aarhus University, Langelandsgade 140, DK-8000 Aarhus C, Denmark

[5]Institute for Atmospheric and Earth System Research/Physics, Faculty of Science, University of Helsinki, P. O. Box 64, FIN-00014 Helsinki, Finland

[6]pi-numerics, Wallbachsiedlung 28, 5202 Neumarkt am W., Austria

[7]Department of Earth and Environmental Science, University of Pennsylvania, Philadelphia, PA, USA 19104-6316

[#]Z.H.F. and F.F.M. contributed equally.

*Corresponding e-mail: hbxie@dlut.edu.cn; niewei@nju.edu.cn; frjoseph@sas.upenn.edu; putian.zhou@helsin-ki.fi

Totally, 23 pages, 10 figures, 3 tables

Supporting Information

Contents

Computational details for fractional yields calculation S3
Box Modeling. S3
Comparison of the formation of $P_{TH-1-1-2}$ and $IM_{TH-1-1-1}$. S10
Discussion about the energies of RCs and TSs S11
Table S2 ....S12
Table S3 ....S12
Figure S1. ....S13
Figure S2. ....S14
Figure S3. ....S15
Figure S4. ....S16
Figure S5. ....S18
Figure S6. ....S18
Figure S7. ....S19
Figure S8. ....S20
Figure S9. ....S21
Figure S10. ....S22
References S23

Supporting Information

Computational details for fractional yields calculation

The master equation (ME) approach was used for describing the nonequilibrium kinetics involving various reaction complexes (wells) on the potential energy surfaces (PES). The ME approach has been discussed in previous studies,[1] and only a brief presentation is given here. Briefly, the population distribution for the reactants and intermediate on the PES is calculated as a function of time by solving a set of differential equations that describe collisional energy transfer within species and interconversion between species. The general form of the ME used in this study is

$$\frac{d}{dt}p = Mp$$

where **p** is a vector, describing the time evolution populations, and **M** is a collision matrix that describes the rate of population transfer due to collisional energy transfer and reaction. In solving the master equation, a matrix describing the population evolution within and between potential energy wells was diagonalized, yielding a set of eigenvalues.

Box Modeling.

1. All considered reaction pathways for toluene oxidation.

The original mechanism (denoted by "Ro") here refers to the chemistry scheme directly generated by MCM v3.3.1.[2] In this mechanism, toluene is oxidized by ·OH to produce TLBIPEROOH and TLBIPERNO3 after several steps, which can be further oxidized by ·OH as shown below:

TLBIPEROOH + ·OH = TLOBIPEROH + ·OH: 9.64×10^{-11} [Ro1]

TLBIPERNO3 + ·OH = TLOBIPEROH + NO_2: 7.16×10^{-11} [Ro2]

In the new mechanism (denoted by "Rn"), a new oxidation pathway has been proposed in this study. And the above two reactions are replaced by the following reactions:

TLBIPEROOH + ·OH = TEPOX + ·OH: $8.32 \times 10^{-10} \times 0.561$ [Rn1]

TLBIPEROOH + ·OH = $C_7H_{10}O_5$: $8.32 \times 10^{-10} \times 0.0144$ [Rn2]

TLBIPEROOH + · OH = 0.00998MGLYOX + 0.00998$C_4H_6O_5$ + 0.323$C_4H_6O_3$ + 0.323 $C_3H_4O_3$ + 0.000420IMTH1O2ROOH + 0.0906IMTH1O2RONO2: $8.32 \times 10^{-10} \times 0.424$ [Rn3]

TLBIPEROOH + OH = $IM_{TH-2-O2}$: 8.32 × 10^{-10} × 0.0006 [Rn4]

TLBIPERNO3 + ·OH = MGLYOX + $C_4H_5O_6N$: 6.53 × 10^{-10} × 0.560 [Rn5]

TLBIPERNO3 + ·OH = $C_7H_9O_8N$ + HO_2·: 6.53 × 10^{-10} × 0.214 [Rn6]

TLBIPERNO3 + ·OH = TEPOX + NO_2: 6.53 × 10^{-10} × 0.224 [Rn7]

TLBIPERNO3 + ·OH = $C_7H_9O_7N$: 6.53 × 10^{-10} × 0.002 [Rn8]

TEPOX + ·OH = TEPOX_ox: 3.03 × 10^{-11} [Rn9]

TEPOX_aq = TEPOX-H^+: 5 × 10^{-2} * [H^+] [Rn10]

The numbers at the end of equations are the reaction rate coefficients (cm^3 $molecule^{-1}$ s^{-1} or s^{-1}) and each reaction is tagged with an index. The reaction rate of Rn9 was calculated with chemical quantum method. For aqueous reactions of TEPOX (Rn10), the aqueous second-order reaction rate constant value 5 × 10^{-2} M^{-1} s^{-1} from corresponding IEPOX reaction[3] was employed, due to their similar structures and aqueous reaction mechanism (see Figure S9 and Reference 4). The reaction rate of TEPOX changes with pH value as shown in Rn10. In addition, pH has a possibility to affect the second-order rate constants by changing the distribution of different forms. According to the calculated forms of TEPOX as a function of pH (see Figure S8), the TEPOX remains in neutral form at the typical aerosol pH ranges (1-5). Therefore, the effect of pH on the aqueous second-order reaction rate constant could be negligible. The properties, including the chemical formula, molar mass and saturation vapor pressure (SVP), of the newly added species are shown in Table M_SVP (see details in spreadsheet). In addition, the aqueous phase reaction of TEPOX is also included (Rn10), where TEPOX_aq means TEPOX condensed in the particle aqueous phase. For the aqueous phase reaction of TEPOX, the reaction rate coefficient (s^{-1}) is dependent on the molarity of hydrogen ion [H^+] (mol L^{-1}), which can be calculated from the pH value:

$$[H^+] = 10^{(-pH)}$$

where pH (dimensionless) value is an input in the simulations.

2. Model setup

In order to evaluate the impacts of different factors on the SOA formation, various simulation cases were conducted. The environment parameters, including air temperature, air pressure, relative humidity (RH) and actinic fluxes, are the same for all the simulation cases. Among them, the air temperature, air pressure and RH are set as constants, which is 300 K, 1 atm (1.01325×10^5 Pa) and 70%, respectively. In current model, RH only affects the number concentration of water vapor (H_2O). The actinic fluxes were derived from the Table 3.7 in Finlayson-Pitts and Pitts,[3] which reflected a diurnal cycle at the Earth's surface according to the changing of solar zenith angle. All the simulations were conducted for two days with a time step of 5 seconds.

In the base case, typical values and default configuration were applied as a control run, which shows a typical summertime urban environment. For example, the mixing ratios of molecular hydrogen (H_2), ozone (O_3), nitric oxide (NO), nitrogen dioxide (NO_2), sulfur dioxide (SO_2), carbon monoxide (CO), nitrous acid (HONO), methane (CH_4) and toluene are set to 600 parts per billion in volume (ppbv), 60 ppbv, 0.1 ppbv, 1 ppbv, 6 ppbv, 1300 ppbv, 1 ppbv, 2000 ppbv and 5 ppbv, respectively (Table M_SIM, see details in spreadsheet). Their mixing ratios are kept constant as the input in the calculation of chemistry scheme. Considering the computation efficiency, not all the organic compounds are considered to be condensable, only the ones whose SVP under a threshold are assumed to condense. In the base case, the threshold of SVP is 1.0×10^{-7} atm, namely about 0.01 Pa.

3. Analysis of sensitivity simulations

Based on different chemistry schemes, we conducted five sets of sensitivity simulations:

(1). Set base: Original mechanism (prefixed by base)

(2). Set new: New mechanism without considering the oxidation of TEPOX and its aqueous phase reaction, namely, excluding Rn9 and Rn10 reactions (prefixed by new)

(3). Set new_aq: New mechanism without considering the oxidation of TEPOX, namely, excluding Rn9 reaction (prefixed by new_aq)

(4). Set new_ox: New mechanism without considering the aqueous phase reaction of TEPOX, namely, excluding Rn10 reaction (prefixed by new_ox)

(5). Set new_ox_aq: Full new mechanism (prefixed by new_ox_aq)

In each set of sensitivity simulation, several input parameters or configurations have been altered to evaluate their individual impacts on simulation results, especially on the SOA mass yield (Y_{mass}). For example, the mixing ratios of NO_2, SO_2, HONO and toluene are in the range of 2 ppbv - 50 ppbv, 3 ppbv - 20 ppbv, 0.3 ppbv - 5 ppbv and 0.5 ppbv - 10 ppbv, respectively. It should keep in mind that the mixing ratio of NO is always kept as one tenth of NO_2. The SVP threshold for condensable vapors varied from 1.0×10^{-6} atm to 1.0×10^{-10} atm, and pH value in the simulations with aqueous reaction varied from 1 to 5. Furthermore, some special cases are also included. For example, in the 'TOLUENE_V5' cases, the mixing ratio of toluene starts at 5 ppbv and then reacts over time rather than keeping constant. In the 'SVP_EPI' cases, the SVP values of newly added species in the new mechanism are calculated by the EPI suite software, while in other cases, their SVP values are calculated by the SIMPOL method. In the 'TH_Hn' cases, the SVP value of TEPOX-H^+ is set to several orders (indicated by the number n) lower than that of TEPOX instead of 10^{-20} atm (Table M_SVP). Different configurations can also be combined, e.g., the case 'new_ox_aq_pH2_NO2_H50' means full new mechanism with pH of 2 and NO_2 mixing ratio of 50 ppbv. All the detailed setup of the simulations is shown in SI-Table_M_SVP_and_Table_M_SIM. The impact of individual parameters on Y_{mass} are discussed in following part.

3.1 NO_x

The simulations with varying NO_x (NO + NO_2) mixing ratios show that Y_{mass} decreases as NO_x mixing ratio increases, but the reasons for original and new mechanisms are slightly different. In the original mechanism, MNCATECOOH and MNNCATCOOH contribute about 98% together to Y_{mass} at the end of the simulation in the base case and all the NO_x varying cases (Figure S10a). Both MNCATECOOH and MNNCATCOOH have very low volatilities with $10^{-8.7}$ Pa for MNCATECOOH and 10^{-}

$^{8.9}$ Pa for MNNCATCOOH at 298.15 K (Table M_SVP). As NO_x mixing ratio increases, the contribution of MNNCATCOOH increases from 46.9% in the base case (NO_2 mixing ratio is 1 ppbv) to 83.1% in the NO2_H50 case (NO_2 mixing ratio is 50 ppbv).

From the following reactions, we can find that the MNCATECH is a common precursor (Ro3 and Ro4 reactions). Increasing the mixing ratio of NO_x will produce more MNCATECO and MNNCATECO whose SVP are $10^{-4.1}$ Pa and $10^{-4.5}$ Pa, respectively (Ro5~Ro8 reactions). In addition, as the NO_x mixing ratio increases, the concentration of HO_2 will decrease, e.g., the HO_2 number concentration is about two orders of magnitude lower in NO2_H50 case than that in the base case. Furthermore, the Ro3 and Ro4 reactions have also explained the increasing contribution of MNNCATCOOH as NO_x mixing ratio increases.

$$\text{MNCATECH} + NO_3 = \text{MNNCATECO2} \quad \text{[Ro3]}$$

$$\text{MNCATECH} + \cdot OH = \text{MNCATECO2} \quad \text{[Ro4]}$$

$$\text{MNNCATECO2} + HO_2\cdot = \text{MNNCATCOOH} \quad \text{[Ro5]}$$

$$\text{MNNCATECO2} + NO = \text{MNNCATECO} \quad \text{[Ro6]}$$

$$\text{MNCATECO2} + HO_2\cdot = \text{MNCATECOOH} \quad \text{[Ro7]}$$

$$\text{MNCATECO2} + NO = \text{MNCATECO} + NO_2 \quad \text{[Ro8]}$$

In the new mechanism, MNCATECOOH and MMNCATCOOH, together with several new compounds including TEPOX, IMTH1O2RONO2, $C_7H_9O_8N$ and $C_7H_{10}O_5$ contribute around 90% to Y_{mass} in both new and new_ox cases (Figure S10b and c). However, when NO_x mixing ratio increases, $C_7H_9O_8N$ becomes dominant and contributes more than 90% to Y_{mass} when NO_2 mixing ratio is larger than 20 ppbv in both new and new_ox cases (Figure S10b and c). The reason is that under high NO_x concentration, TLBIPERNO3 is produced much more than TLBIPEROOH, which results in higher yield of $C_7H_9O_8N$ and lower yield of other condensable vapors mentioned above (see reactions Rn1 to Rn8). And compared to other dominant condensable vapors, e.g., MNCATECOOH, MMNCATCOOH, TEPOX and IMTH1O2RONO2, $C_7H_9O_8N$ has higher volatility of $10^{-5.2}$ Pa. Therefore, Y_{mass} also

decreases with the increasing of NO_x mixing ratio in the new mechanism. The explanations also apply similarly in the new_aq and new_ox_aq cases (Figure S10d and e).

3.2 SO_2, HONO and toluene

In all the SO_2 cases except in the new_ox_aq case set, Y_{mass} only decreases less than 1% when SO_2 mixing ratio increases from 3 ppbv to 20 ppbv within each set of simulations (Table M_SIM). The inverse relationship results from the competition of SO_2 for ·OH, which causes a slight decrease production of condensable vapors. In the new_ox_aq case set, the decrease of OH could also increase the amount of TEPOX_aq which increases the Y_{mass} from TEPOX, so the Y_{mass} is lower (less than 1%) in the case new_ox_aq compared to new_ox_aq_SO2_L3 and new_ox_aq_SO2_H20. In contrary, the increasing HONO mixing ratio will increase ·OH concentration, resulting in higher Y_{mass} (Table M_SIM). For example, the ·OH concentration is about twice in the HONO_H5 case in the middle of the second day as that in the base case, causing about 85% increase of Y_{mass}. The results are similar in the new and new_aq sets. In the new_ox and new_ox_aq sets, the increasing ratio of ·OH concentration in HONO_H5 case is the same but the increasing ratio of Y_{mass} is lower, which is about 53%. This is due to the ·OH oxidation of TEPOX (Rn9 reaction) which consumes ·OH but does not produce condensable vapors.

In the base set, Y_{mass} is lower when the toluene mixing ratio is higher, because the production of condensable vapors is smaller than the additionally reacted toluene. In the TOLUENE_V5 case, the toluene is consumed and thus the reacted toluene is lower than the base case, which results in a higher Y_{mass} (Table M_SIM). While in the new and new_aq sets, Y_{mass} is larger at higher toluene mixing ratios due to the increased production of TEPOX (Figure S10b and d). Therefore, Y_{mass} in the TOLUENE_V5 is lower than the standard cases new and new_aq. In the new_ox and new_ox_aq sets, the situation is more complicated because the additionally produced TEPOX is not only condensing but only oxidized by ·OH at higher toluene mixing ratios. So these two compensated effects result in decreasing of Y_{mass} when toluene mixing ratio is

increasing. Otherwise, the relationship between Y_{mass} and toluene mixing ratio is similar with the base set (Figure S10c and e).

3.3 SVP

Considering the computation efficiency, not all the species are considered as condensable vapors although some of them may also condense under certain conditions. In the sensitivity simulations, different SVP thresholds are tested to evaluate the impact of the threshold choice. It should be noted that the lower the SVP threshold is, the less condensable species are included in the condensable vapor list (Table M_SVP). The results show that when the threshold of SVP is set to 1×10^{-10} atm, Y_{mass} decreases dramatically since only a few species are condensing. However, the difference between 1×10^{-7} atm and 1×10^{-6} atm is less than 1% for all the sets (Table M_SIM). So using 1×10^{-7} atm as a standard threshold of SVP is a reasonable choice in this study.

Different methods of calculating SVP values sometimes show large difference for some species. In order to evaluate this uncertainty in the new mechanism, we applied the SVP values calculated by EPI suite software[5] in the SVP_EPI cases. Table M_SVP shows that the SVP values calculated from EPI suit are about one order of magnitude higher than that from SIMPOL method[6]. Therefore, Y_{mass} is about 40% lower in the new (37.7%) and new_aq (38.6%) sets, and about 10% lower in the new_ox (10.1%) and new_ox_aq (13.7%) sets. This indicates that the importance of the accurate estimation of the SVP values in calculating the Y_{mass}. In the TH_Hn (n = 1, 3, 5) cases, the impact of the SVP value of TEPOXH_ion has been evaluated for new_aq and new_ox_aq sets. The results show that as long as TEPOXH_ion has more than one order of magnitude lower of SVP than TEPOX, Y_{mass} is not affected (Figure S10d and e).

3.4 pH

In the new_aq and new_ox_aq sets with the aqueous phase reaction, Y_{mass} shows large increasing when pH decreases (Table M_SIM). This is also expected since more TEPOX in the aqueous phase are converted to TEPOXH_ion which is nearly non-volatile, and then the aqueous TEPOX pulls more gaseous TEPOX to the particle phase. Therefore, under low pH conditions, TEPOXH_ion is a dominant contribution to Y_{mass}

(Figure S10d and e). This indicates that the new mechanism with the aqueous phase reaction can play an important role in the acidic urban environment condition.

Comparison of the formation of $P_{TH\text{-}1\text{-}1\text{-}2}$ and $IM_{TH\text{-}1\text{-}1\text{-}1}$.

As presented in the Figure S2b, the energy barrier of forming $IM_{TH\text{-}1\text{-}1\text{-}1}$ via $TS_{TH\text{-}1\text{-}1\text{-}1}$ (8.5 kcal mol^{-1}) is lowest among the considered reaction pathways for the reactions of $IM_{TH\text{-}1\text{-}1}$. However, the $IM_{TH\text{-}1\text{-}1\text{-}1}$ is thermodynamically unstable with a reversible energy barrier of only 0.1 kcal mol^{-1}, and therefore the pathway is reversible. Besides, as a C-centered radical, the $IM_{TH\text{-}1\text{-}1\text{-}1}$ can barrierlessly combine with O_2 to form peroxy radicals ($IM_{TH\text{-}1\text{-}1\text{-}1}$-$RO_2$). Therefore, pathway involving $IM_{TH\text{-}1\text{-}1\text{-}1}$ can be described as

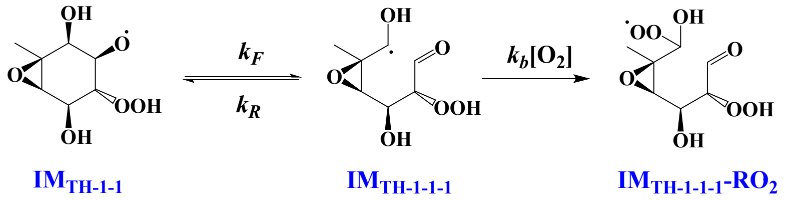

Additionally, we noted that forming $P_{TH\text{-}1\text{-}1\text{-}2}$ via $TS_{TH\text{-}1\text{-}1\text{-}2}$ with the energy barrier of 11.9 kcal mol^{-1} is the second most favorable and the thermodynamics is favorable. Therefore, it is necessary to compare the reaction rate constants of these two reaction pathways to clearly identify the most feasible pathway. Due to the second-step reactions of $IM_{TH\text{-}1\text{-}1\text{-}1}$, it is hard to directly compare the reaction rate of forming $IM_{TH\text{-}1\text{-}1\text{-}1}$ and forming $P_{TH\text{-}1\text{-}1\text{-}2}$. Therefore, we calculated the effective rate ($k_{b,eff}$, in s^{-1}) for the formation of $IM_{TH\text{-}1\text{-}1\text{-}1}$-$RO_2$ as

$$k_{b,eff} = \frac{k_F k_b[O_2]}{k_R + k_b[O_2]}$$

where k_F, k_R, and $k_{b[O2]}$ are rate constants for the reaction of $IM_{TH\text{-}1\text{-}1} \rightarrow IM_{TH\text{-}1\text{-}1\text{-}1}$, $IM_{TH\text{-}1\text{-}1\text{-}1} \rightarrow IM_{TH\text{-}1\text{-}1}$ and $IM_{TH\text{-}1\text{-}1\text{-}1} \rightarrow IM_{TH\text{-}1\text{-}1\text{-}1}$-$RO_2$, respectively. The $k_{b,eff}$ value was estimated to be 2.1×10^2 s^{-1} ($IM_{TH\text{-}1\text{-}1} \rightarrow IM_{TH\text{-}1\text{-}1\text{-}1}$-$RO_2$) based on our calculated values for k_F (4.7×10^6 s^{-1}) and k_R (1.2×10^{12} s^{-1}), and $k_b = 10^{-11}$ cm^3 $molecule^{-1}$ s^{-1} and $[O_2] = 5 \times 10^{18}$ molecules cm^{-3}. We found that the $k_{b,eff}$ value is much lower than that of forming $P_{TH\text{-}1\text{-}1\text{-}2}$ (1.4×10^5 s^{-1}). Therefore, forming $P_{TH\text{-}1\text{-}1\text{-}2}$ is much more favorable than forming $IM_{TH\text{-}1\text{-}1\text{-}1}$ for the reactions of $IM_{TH\text{-}1\text{-}1}$.

Discussion about the energies of RCs and TSs

As shown in the Figure S2a and 4a, the energies of three RCs (RC_{TH-1}, RC_{TH-2} and RC_{TN-2}) are higher than that of the corresponding TSs (TS_{TH-1}, TS_{TH-2} and TS_{TN-2}). Therefore, it is interesting to discuss why these apparent anomalies occur. Firstly, we checked the wavefunction stability of these RCs with the keyword 'stable' in the optimization process, and found that the wavefunction of three RCs are stable. Secondly, by further analysing the energies of RCs and TSs at the M06-2X/6-31+G(d,p) level of theory, we found that the energies of RCs (RC_{TH-1} -6.00 kcal mol^{-1}, RC_{TH-2} -6.25 kcal mol^{-1} and RC_{TN-2} -5.82 kcal mol^{-1}) are lower than that of the corresponding TSs (TS_{TH-1} -3.65 kcal mol^{-1}, TS_{TH-2} -3.84 kcal mol^{-1}, TS_{TN-2} -3.51 kcal mol^{-1}) (Table S1), indicating the IRC calculation is correct. We also test these three pathways using a larger basis set aug-cc-pVTZ within M06-2X functional. It was found the wavefunction of RCs are still stable and the energies of RCs (RC_{TH-1} -6.03 kcal mol^{-1}, RC_{TH-2} -5.20 kcal mol^{-1} and RC_{TN-2} -4.88 kcal mol^{-1}) are also lower than that of the corresponding TSs (TS_{TH-1} -2.54 kcal mol^{-1}, TS_{TH-2} -2.75 kcal mol^{-1} and TS_{TN-2} -2.70 kcal mol^{-1}) at the M06-2X/aug-cc-pVTZ level of theory. In addition, the barrier from RCs to TSs is low at the optimization level, indicating a fast reaction. Generally, the higher level single point energy calculation can change the barrier several kcal mol^{-1}. In addition, RCs and TSs geometry structure at the optimization level could not be the real minima at the single point energy level since the potential energy surface could change with employed the computational level. Therefore, it is single point energy calculation that leads to apparent anomalies in physics. The similar phenomenon was found in our previous studies.[7-10] Actually, it is a barrierless reaction from RCs to TSs at the level of single point energy calculation, still indicating a fast reaction.

Supporting Information

Table S1. Calculated energies (kcal mol^{-1}) of pre-complexes (RCs) and transition states (TSs) at the M06-2X/6-31+G(d,p) level of theory.

M06-2X/6-31+G(d,p)			
Species	ΔE	Species	ΔE
RC_{TH-1}	-6.00	TS_{TH-1}	-3.65
RC_{TH-2}	-6.25	TS_{TH-2}	-3.84
RC_{TN-2}	-5.82	TS_{TN-2}	-3.51
M06-2X/aug-cc-pVTZ			
Species	ΔE	Species	ΔE
RC_{TH-1}	-6.03	TS_{TH-1}	-2.54
RC_{TH-2}	-5.20	TS_{TH-2}	-2.75
RC_{TN-2}	-4.88	TS_{TN-2}	-2.70

Table S2. Lennard-Jones parameters of intermediates for various reactions used in the MESMER simulations.

Species	σ/(Å)	ε/(K)
IM_{TH-1} and IM_{TH-2}	6.83	822
IM_{TN-1} and IM_{TN-2}	6.91	894
$IM_{TH-1-O-1}$	7.11	906

Table S3. Effects of ΔE_d (cm^{-1}) and grain size on the yields of the important species for the reactions of T-ROOH/RONO$_2$ + ·OH.

Reactions	Yields					
	ΔE_d	Grain size = 50 cm^{-1}				Grain size = 25 cm^{-1}
	Species	150	200	250	300	200
T-ROOH + ·OH	$P_{TH-1-1-2}$	2.20%	1.44%	1.05%	0.813%	1.44%
	TEPOX	56.1%	56.1%	56.1%	56.0%	56.1%
	$IM_{TH-1-O2}$	41.7%	42.4%	42.8%	43.1%	42.4%
	$IM_{TH-2-O2}$	0	0.0600%	0.0050%	0.0870%	0.0600%
T-RONO$_2$ + ·OH	$P_{TN-1-1-1}$	0.400%	0.200%	0.200%	0.100%	0.200%
	TEPOX	27.1%	22.4%	18.9%	16.4%	22.4%
	$IM_{TN-1-O2}$	55.9%	56.0%	56.1%	56.1%	56.0%
	$IM_{TN-2-O2}$	16.6%	21.4%	24.8%	27.4%	21.4%

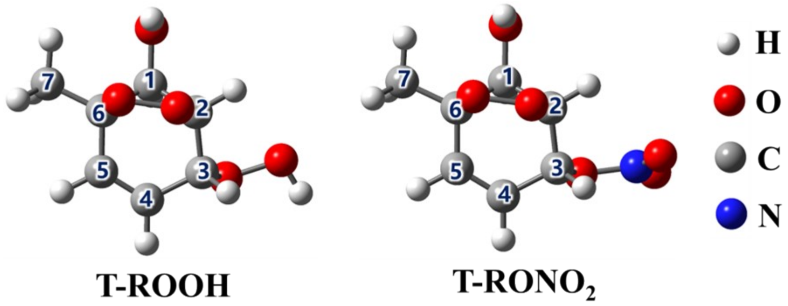

Figure S1. Global minimum of T-ROOH and T-RONO$_2$ obtained at ROCBS-QB3//M06-2X/6-31+G(d,p) level. The number represents the label of the carbon atom in the model compounds.

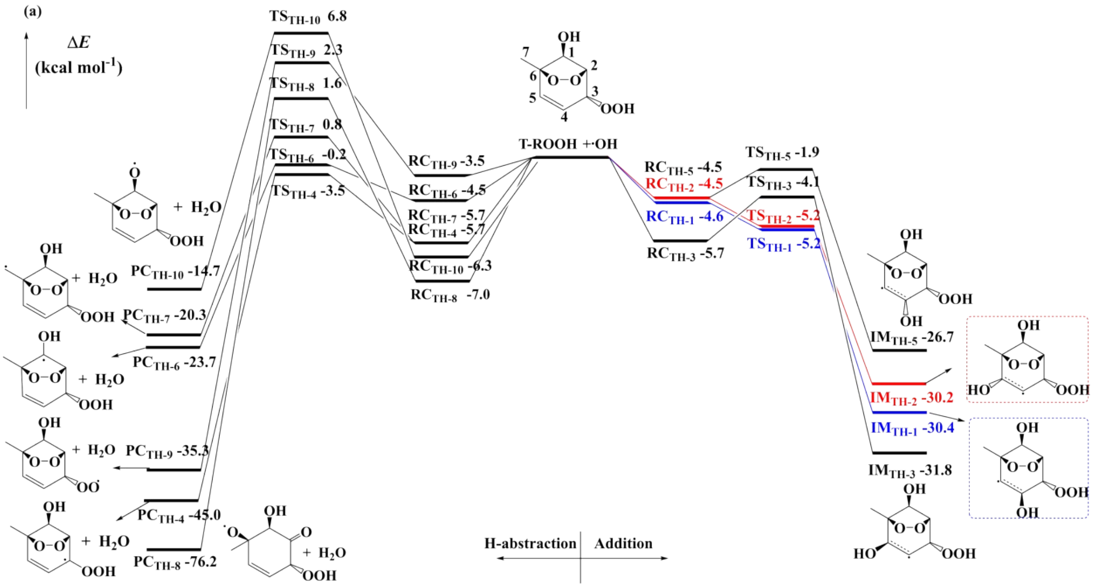

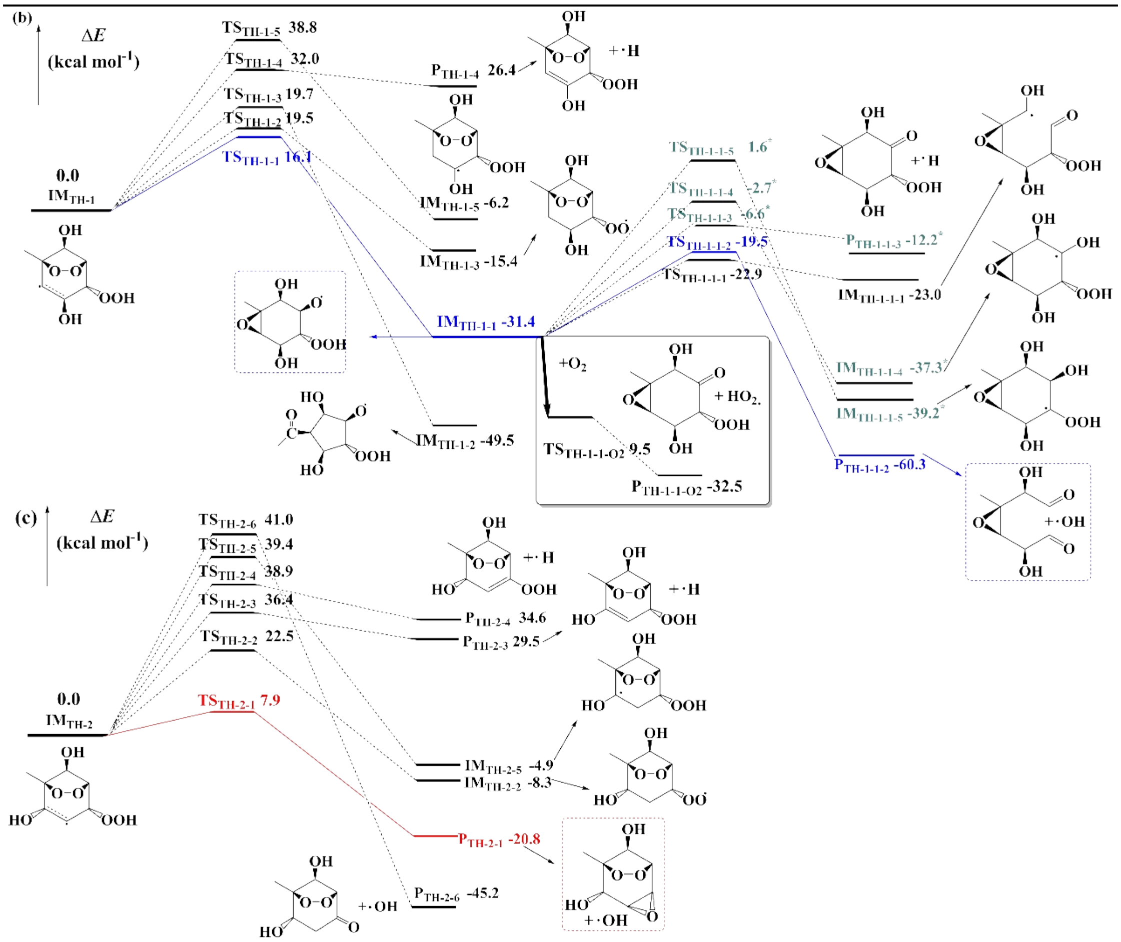

Figure S2. Schematic ZPE-corrected potential energy surfaces for initial reactions of T-ROOH + ·OH (a) and unimolecular reactions of IM_{TH-1} (b) and IM_{TH-2} (c) at the ROCBS-QB3//M06-2X/6-31+G(d,p) level. The symbols RC_{TH-m}, TS_{TH-m}, IM_{TH-m} and P_{TH-m} represent the pre-reactive complex, transition states and intermediates, respectively. It should be noted that the energies of IM_{TH-1-1} + O_2 are set to zero for corresponding reactions in the insert. The symbol '*' stands for the energies used here is obtained from the optimization level.

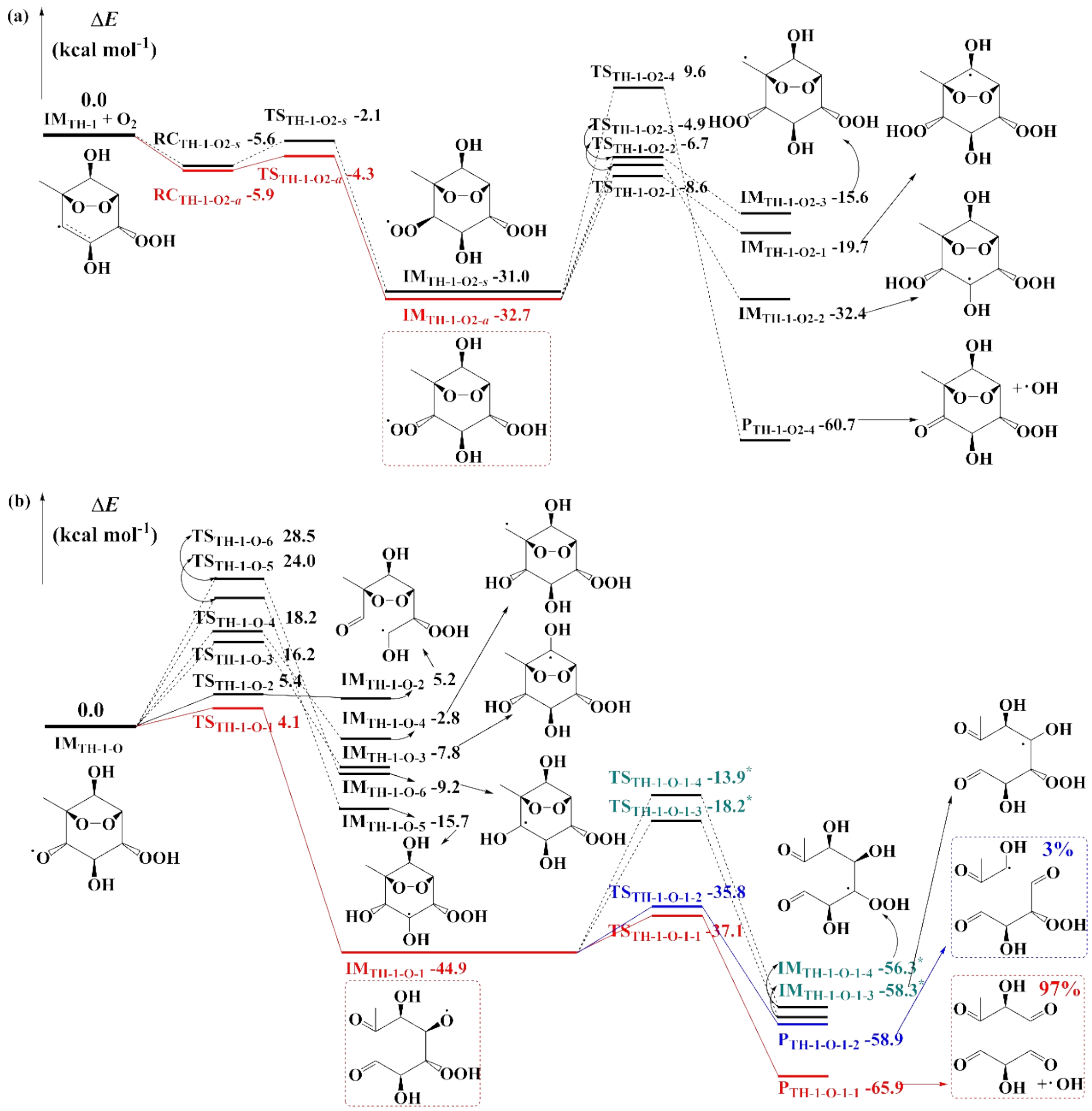

Figure S3. Schematic ZPE-corrected potential energy surfaces for IM_{TH-1} with O_2 (a) and unimolecular reactions of alkoxy radicals IM_{TH-1-O} (b) stating from T-ROOH + ·OH at the ROCBS-QB3//M06-2X/6-31+G(d,p) level. The symbols s and a stand for *syn*- and *anti*-directions of O_2-additions (the same and opposite sides to the -O-O- bridge ring). The symbol '*' stands for the energies used here is obtained from the optimization level.

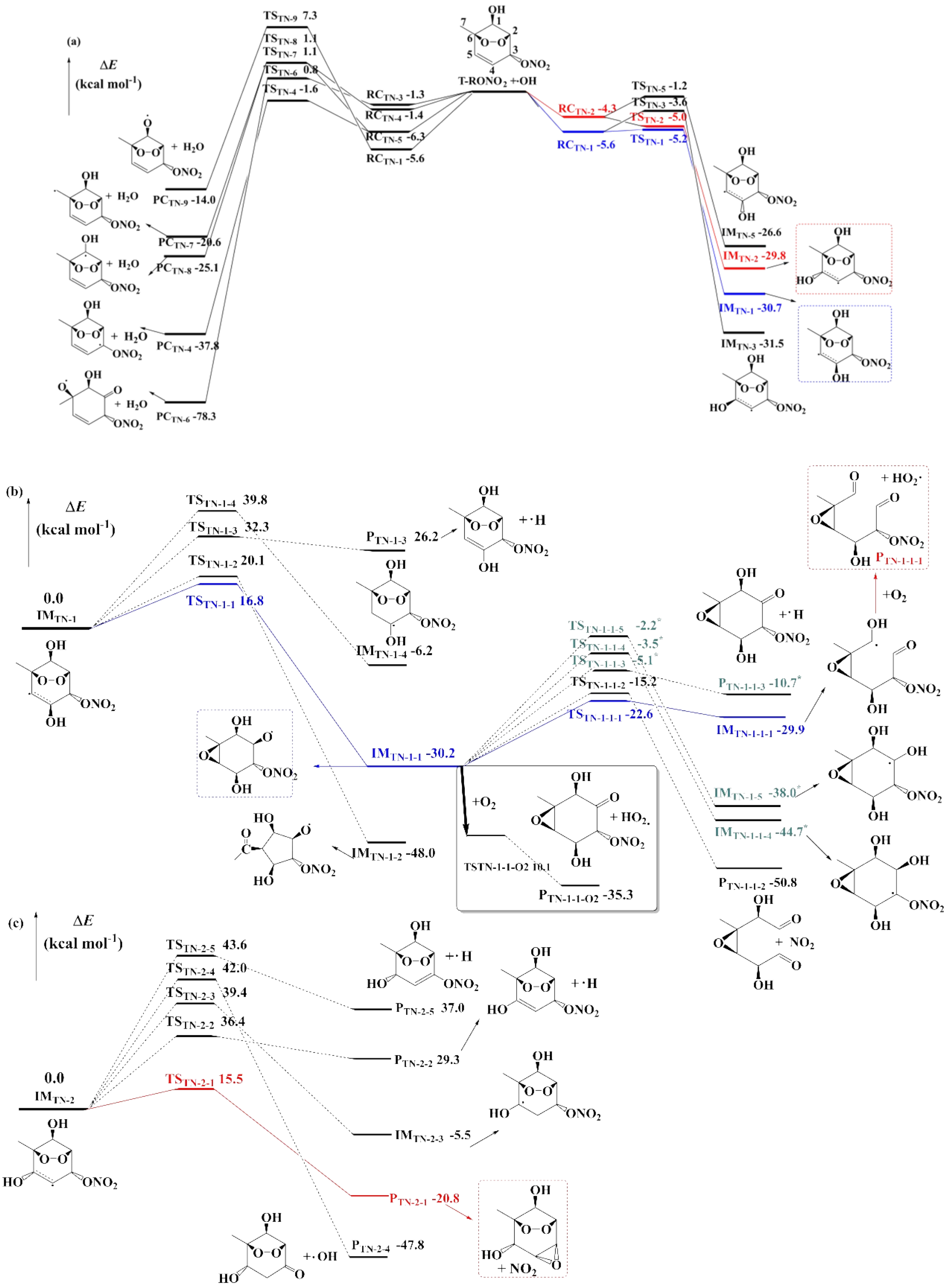

Figure S4. Schematic ZPE-corrected potential energy surfaces for initial reactions of T-RONO$_2$ + ·OH (a) and unimolecular reactions of IM$_{TN\text{-}1}$ (b) and IM$_{TN\text{-}2}$ (c) at the ROCBS-QB3//M06-2X/6-31+G(d,p) level. It should be noted that the energies of IM$_{TN\text{-}1\text{-}1}$ + O$_2$ are set to zero for corresponding reactions in the insert. The symbol '*' stands

for the energies used here is obtained from the optimization level.

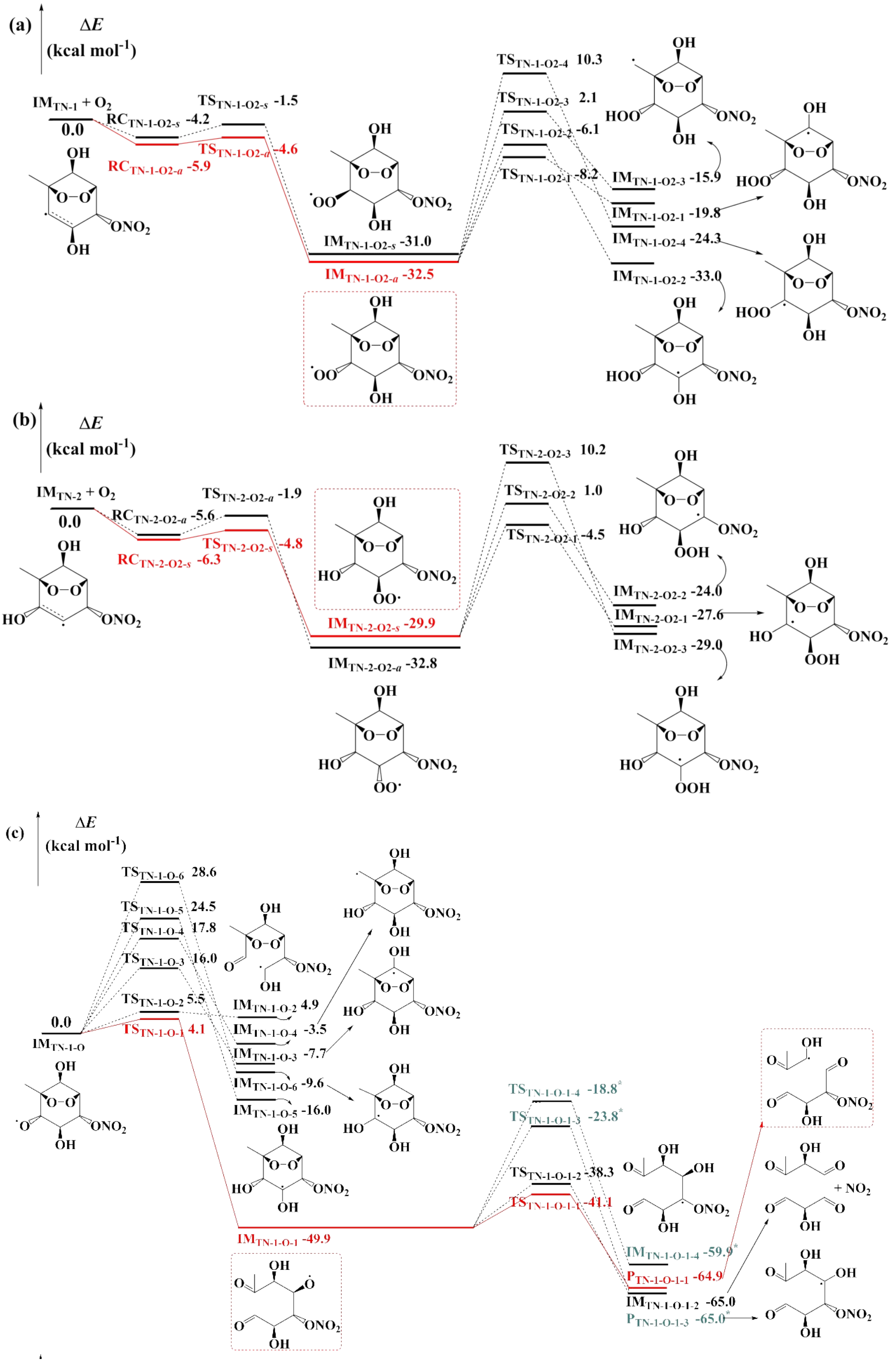

Figure S5. Schematic ZPE-corrected energy surfaces for the reactions of IM_{TN-1} (a) and IM_{TN-2} (b) with O_2 and unimolecular reactions of alkoxy radicals IM_{TN-1-O} (c) and IM_{TN-2-O} (c) stating from T-$RONO_2$ + ·OH at the ROCBS-QB3//M06-2X/6-31+G(d,p) level of theory. *s* and *a* stand for *syn*- and *anti*-directions of O_2-additions (the same and opposite sides to the -O-O- bridge ring). The symbol '*' stands for the energies used here is obtained from the optimization level.

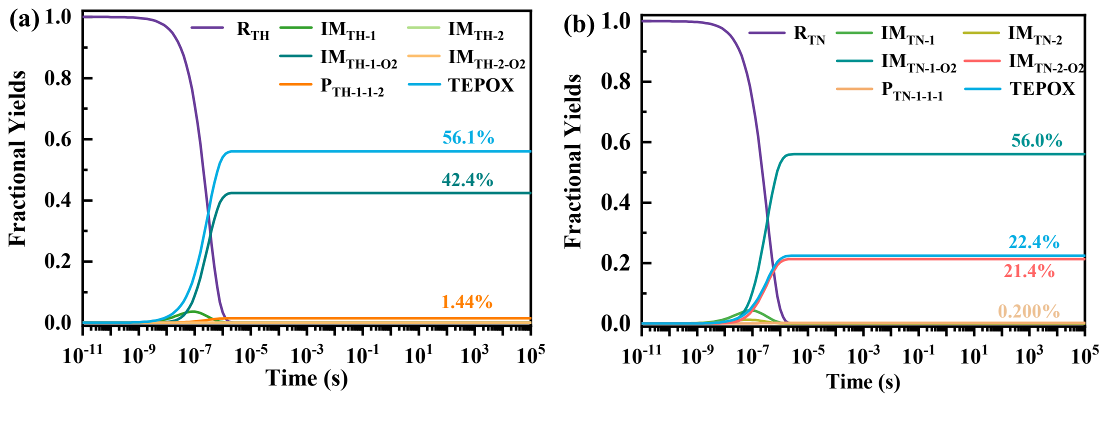

Figure S6. Variation in fractional yields of reaction products for the reactions of T-ROOH (a) and T-$RONO_2$ (b) with ·OH, with reaction time at 298 K and 1 atom.

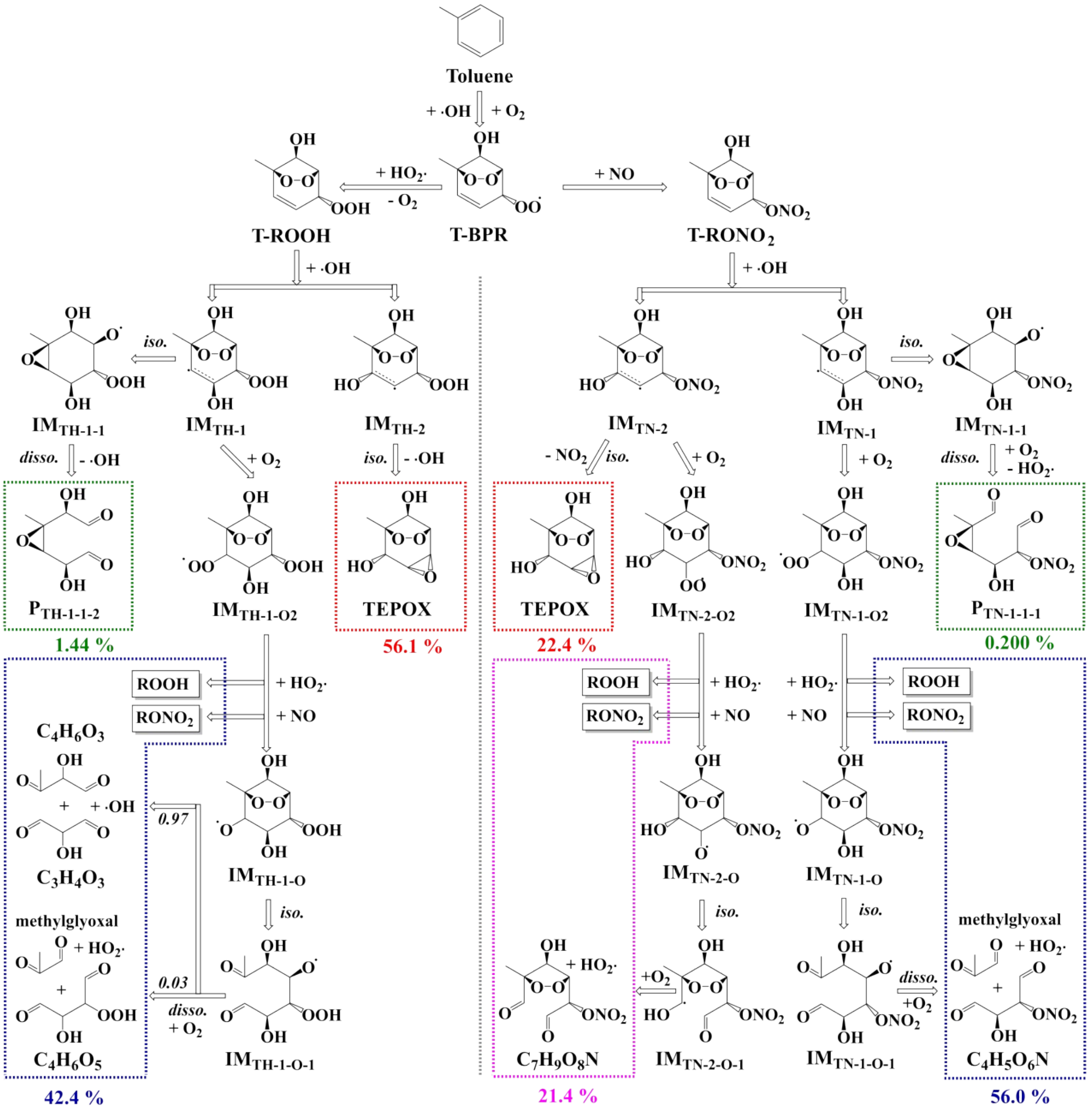

Figure S7. Main atmospheric oxidation pathways and products of the ·OH initiated T-ROOH/T-$RONO_2$ reactions. The calculated fractional yields of the corresponding products starting from T-ROOH/T-$RONO_2$ + ·OH are shown in percentages. The calculated fractional yields of the products starting from $IM_{TH-1-O-1}$ are presented in italic numbers.

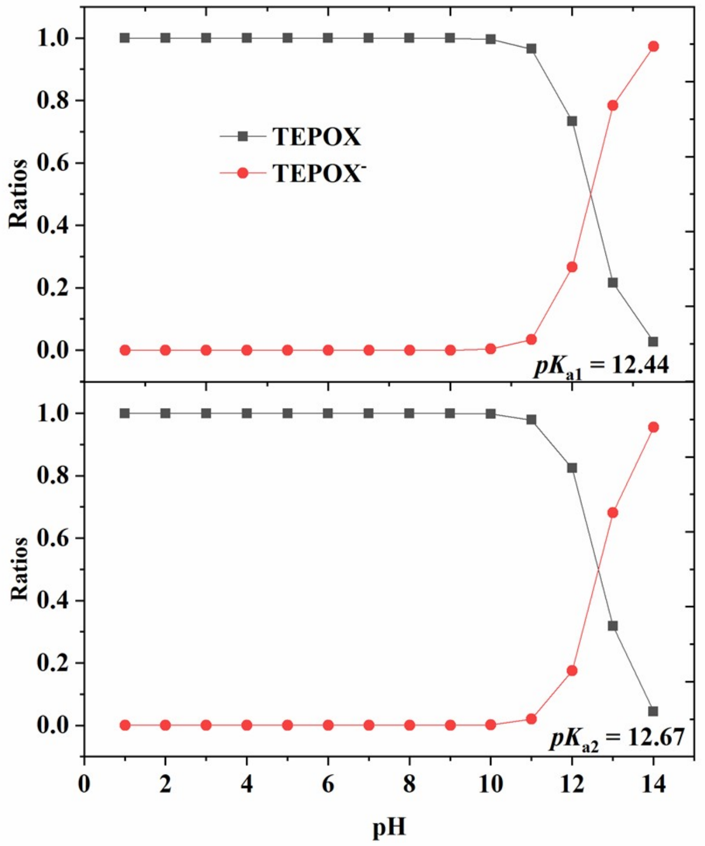

Figure S8. Calculated ratios of different dissociation forms of TEPOX as a function of pH based on the predicted pK_a values (Note: pK_a values of TEPOX were calculated by Graph-pK_a model[11])

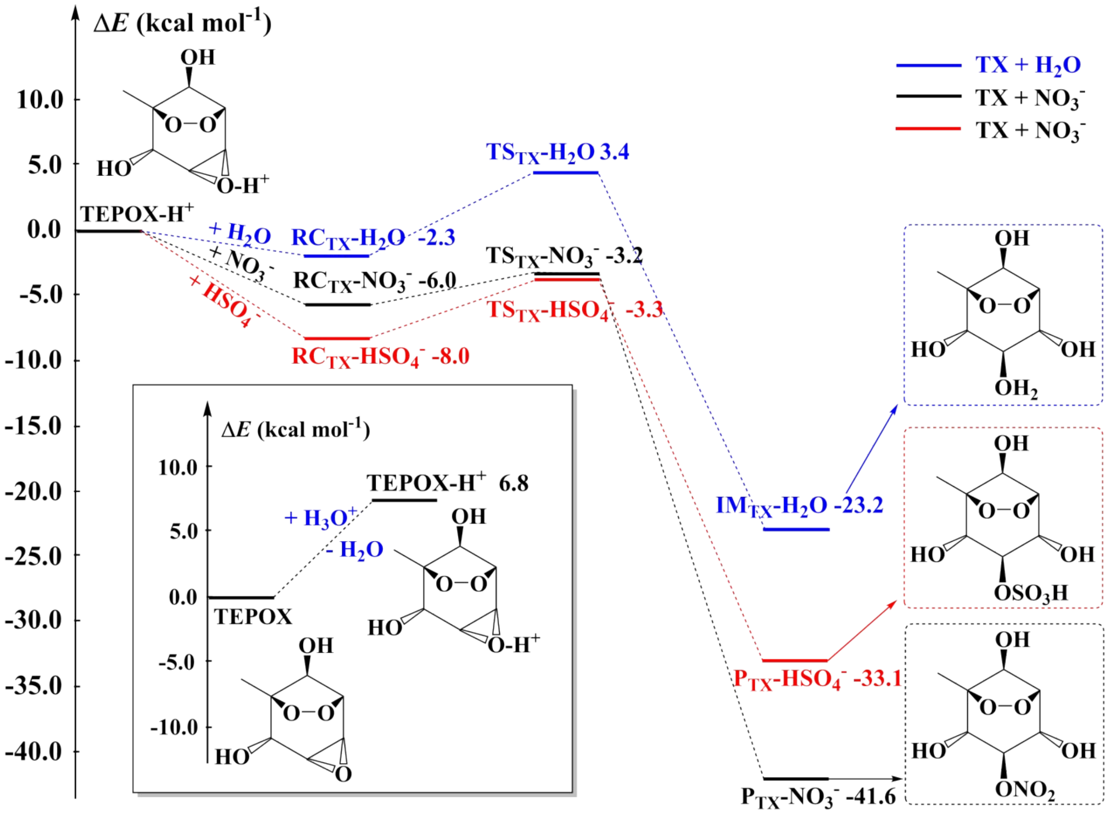

Figure S9. Calculated schematic potential energy surfaces for reactions of protonated TEPOX with H_2O, NO_3^- and HSO_4^- in the aerosol phase at the ROCBS-QB3//M06-2X/6-31+G(d,p) level. The protonation process of TEPOX is shown in the inset.

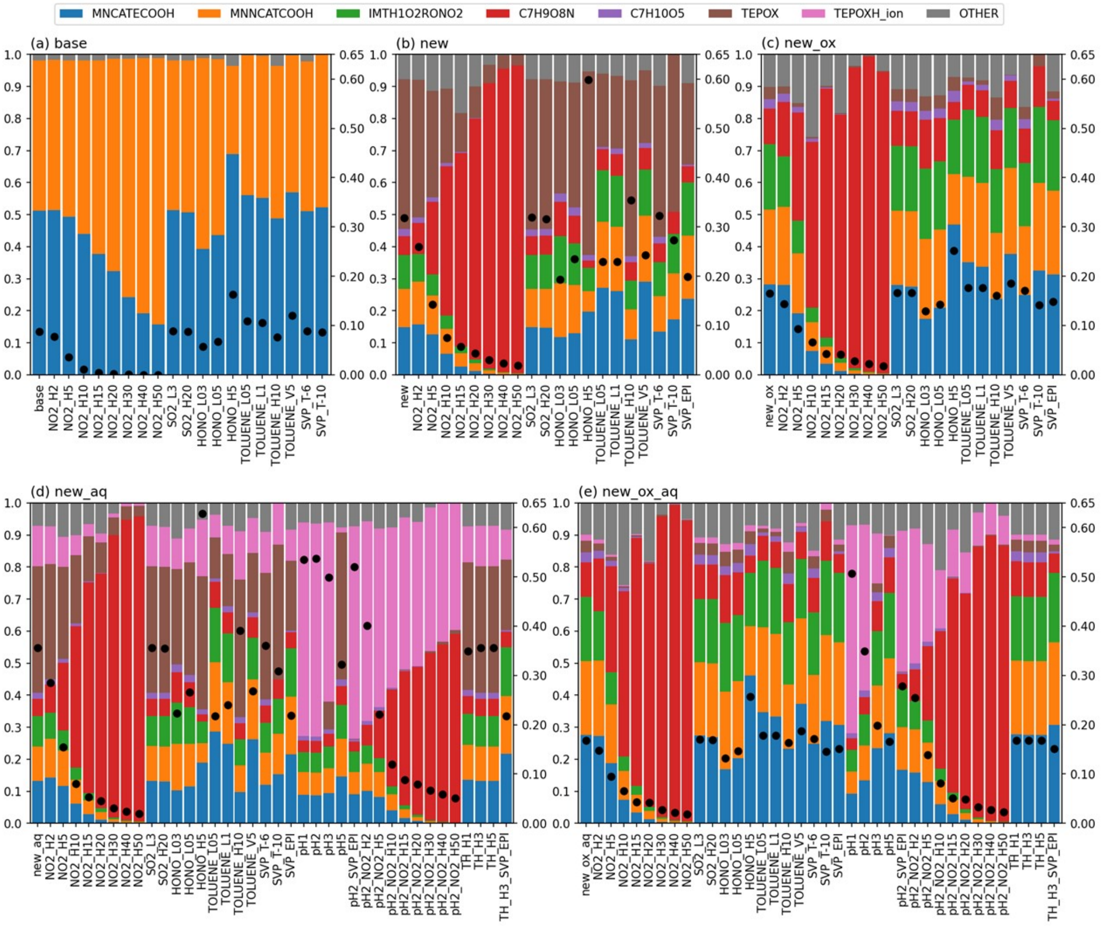

Figure S10. Contributions of seven most contributing species and other species as a whole (OTHER, gray) at the end of individual simulations are plotted. The seven species include MNCATECOOH (blue), MNNCATCOOH (orange), IMTH1O2RONO2 (green), $C_7H_9NO_8$ (red), $C_7H_{10}O_5$ (purple), TEPOX (brown) and TEPOXH_ion (pink). The Y_{mass} at the end of each simulation is also plotted with a black dot. Each set of simulations, including (a) base, (b) new, (c) new_ox, (d) new_aq and (e) new_ox_aq, are plotted in different subplots. The prefixes in case names in the sets of new, new_ox, new_aq and new_ox_aq are not shown for clarity.

References

1. M. A. Blitz, K. J. Hughes, M. J. Pilling and S. H. Robertson, *J. Phys. Chem. A*, 2006, **110**, 2996-3009.
2. M. E. Jenkin, S. M. Saunders, V. Wagner and M. J. Pilling, *Atmos. Chem. Phys.*, 2003, **3**, 181-193.
3. N. C. Eddingsaas, D. G. VanderVelde and P. O. Wennberg, *J. Phys. Chem. A*, 2010, **114**, 8106-8113.
4. I. R. Piletic, E. O. Edney and L. J. Bartolotti, *Phys. Chem. Chem. Phys*., 2013, **15**, 18065-18076.
5. U. EPA.
6. J. F. Pankow and W. E. Asher, *Atmos. Chem. Phys.*, 2008, **8**, 2773-2796.
7. J. W. Xue, F. F. Ma, J. Elm, J. W. Chen and H. B. Xie, *Atmos. Chem. Phys.*, 2022, **22**, 11543-11555.
8. H. B. Xie, F. F. Ma, Y. F. Wang, N. He, Q. Yu and J. W. Chen, *Environ. Sci. Technol.*, 2015, **49**, 13246–13255.
9. G. T. Yu, X. R. Huang, Y. H. Ding, C. C. Sun and A. C. Tang, *J. Comput. Chem.*, 2006, **27**, 749-761.
10. J. L. Pang, Y. M. Wang, X. M. Zhang, Y. P. Hua, Z. Wu and Y. H. Ding, *IOP Conf. Ser.: Earth Environ. Sci.*, 2020, **545**, 012023.
11. J. C. Xiong, Z. J. Li, G. C. Wang, Z. Y. Fu, F. S. Zhong, T. Y. Xu, X. M. Liu, Z. M. Huang, X. H. Liu, K. X. Chen, H. L. Jiang and M. Y. Zheng, *Bioinformatics*, 2022, **38**, 792-798.
